# Comparative analysis of pre-operative findings and post-operative outcomes in primary and secondary macular holes at a tertiary eye hospital in South India

**DOI:** 10.1186/s40942-024-00597-7

**Published:** 2024-10-14

**Authors:** Shubham Darade, Rupal Kathare, Ayushi Choudhary, Gaurang Sehgal, Jay Chhablani, Kanika Godani, Naresh Kumar Yadav, Priyanka Gandhi, Prathiba Hande, Rubble Mangla, Vishma Prabhu, Ramesh Venkatesh

**Affiliations:** 1 Dept. of Retina-Vitreous, Narayana Nethralaya, #121/C, Chord Road, 1st R block Rajaji Nagar, Bengaluru, 560010 India; 2 Medical Retina and Vitreoretinal Surgery, University of Pittsburgh School of Medicine, 203 Lothrop Street, Suite 800, Pittsburg, PA 15213 USA

**Keywords:** Macular hole, Idiopathic, Secondary, Outcomes, Macular hole indices

## Abstract

**Purpose:**

This study aimed to compare demographics, clinical characteristics, and post-surgical outcomes between idiopathic and secondary full-thickness macular holes (MHs).

**Methods:**

A retrospective analysis of 348 eyes from 339 patients treated between June 2017 and December 2023 was conducted. The study included both idiopathic and secondary MHs, excluding cases where surgery was not performed or lacked sufficient follow-up. Demographic data, visual acuity (VA), ocular characteristics, and optical coherence tomography measurements were analyzed.

**Results:**

Idiopathic MHs were identified in 308 eyes (89%), and secondary MHs in 40 eyes (11%). Idiopathic MH patients were older (mean age: 68.26 vs. 60.13 years; *p* = 0.001) and more commonly female (63% vs. 40%; *p* = 0.005). Post-surgical closure was achieved in 86% of all MHs, with a median VA improvement of 15 ETDRS letters (3 Snellen lines). However, secondary MHs had a lower closure rate (67% vs. 89%, *p* = 0.001) and less VA improvement (2 lines vs. 3 lines, *p* = 0.001) compared to idiopathic MHs. Significant differences in maximal basal diameter and diameter hole index were noted between the groups.

**Conclusions:**

Secondary MHs, accounting for 11% of surgical cases, show poorer anatomical and visual outcomes than idiopathic MHs. Despite lower success rates, early surgical intervention in secondary MHs is recommended to enhance outcomes. Differentiating between idiopathic and secondary MHs is crucial for optimal management.

## Introduction

A full-thickness macular hole (MH) is a fundus examination finding that involves a central foveal defect extending from the internal limiting membrane to the photoreceptor layer, sparing the retinal pigment epithelium (RPE) [1]. Multiple tractional forces act on the fovea, including inward traction exerted by the vitreous, outward traction exerted by the sclera as in posterior staphyloma, and tangential traction, which exerts a centrifugal force on the fovea via the epiretinal membrane, internal limiting membrane, or the equatorial expansion of the globe [2]. A MH is caused by these tractional forces acting on an inherently weak and structurally deficient fovea, either alone or in combination [2, 3]. MHs either can be secondary MHs, which occur as a consequence to pre-existing ocular pathological condition, or primary or idiopathic MHs, which occurs secondary to physiological changes such as age-related posterior vitreous detachment [4–6]. 

The main difference in the management of an idiopathic MH and a secondary MH is the clinicians’ promptness to recommend surgery in these cases. While prompt vitrectomy is the preferred treatment for idiopathic MHs, secondary MHs are typically managed with observation and deferred vitrectomy [7]. Observation is usually recommended in the management of secondary MHs as there are higher chances of spontaneous closure, to acquire time to treat the cause first, or to abandon any intervention in the event of unsalvageable underlying pathology. The medical literature contains plenty of information on the pathogenesis, surgical techniques, and treatment outcomes of primary or idiopathic MHs; however, MHs caused by secondary ocular pathologies have received little attention [8, 9]. This is despite the fact that the pathogenetic mechanisms responsible for secondary MH formation are similar to those for idiopathic MHs, with some variations, and the surgical steps for managing both types of MH are nearly identical, with a few exceptions [3]. There are a few articles in the literature that independently discuss the surgical outcomes of secondary MHs after myopia, trauma, or pars plana vitrectomy.^6,9–15^ However, there is insufficient evidence in the literature which compares and discusses the demographics, pretreatment MH characteristics, and post-operative surgical outcomes between an idiopathic and a secondary MH. Huang et al. compared and found significant differences in optical coherence tomography (OCT) characteristics between eyes with full-thickness traumatic MHs and full-thickness idiopathic MHs. However, the study did not look at the differences in surgical outcomes between the two groups of patients [10]. 

Given this setting and to fill the literature gap, we retrospectively analyzed and compared idiopathic and secondary full-thickness MHs, focusing on demographics, clinical manifestations, and postsurgical anatomical and functional outcomes.

## Methods

The hospital’s electronic medical system was used to identify full-thickness MHs presenting to the retina clinic of Narayana Nethralaya eye hospital in South India, Bangalore between June 2017 and December 2023. MH caused by other ocular pathologies and idiopathic MH were included. The analysis then excluded cases where surgery was recommended but not performed and where follow-up data of sufficient duration to assess hole closure were unavailable. Thus, only MH cases of any etiology who underwent vitreous surgery (not limited to a single surgeon) and had enough follow-up to comment on MH closure were included for analysis.

After identifying the cases, demographic information like age and gender, ocular details like laterality of the affected eye, pre-operative best-corrected visual acuity (VA), lens status (phakia, pseudophakia, or aphakia), cause of MH development (idiopathic or secondary), whether the MH surgery was performed with concurrent cataract surgery, and post-operative best-corrected VA at the 4-week follow-up interval were recorded. Secondary post-vitrectomy MHs occurred after vitrectomy for reasons unrelated to the primary surgical intervention for MH.

OCT scans of these eyes were obtained from the spectral domain Spectralis (Heidelberg Engineering, Germany) machine to document the MH’s pre and posttreatment characteristics. For noting the measurements and OCT findings, a horizontal line macular volumetric OCT scan over a 6 × 6 mm area and passing through the macula and foveal center and having the largest diameter of MH was selected. On the pretreatment OCT scan, the following qualitative MH features were observed: (1) foveal attachment or detachment of the posterior cortical vitreous; (2) the presence of an epiretinal membrane adjacent to the MH; (3) the presence of intraretinal cystic spaces at the MH edges; and (4) RPE hyperreflectivity at the foveal center. Additional measurements were quantified from the pretreatment OCT scan using the Spectralis software’s built-in line and area measurement tools (Heidelberg Eye Explorer, HEYEX version 1.10.2.0). These included: least inner diameter within the MH, maximal basal diameter, nasal and temporal slopes of the MH, minimum and maximal MH height (all in microns) and MH area (in sq. millimetres). These measurements were used to calculate the MH indices using pre-existing formulas [11]. 

Two masked graders (SD and RK) received 30 randomly selected OCT scans from the study (divided equally between the two groups) to ensure consistency in documenting MH measurements and observations. The two graders’ MH OCT measurements and findings were compared. After a significant agreement in OCT measurements and findings related to MHs, each grader independently evaluated the remaining OCT scans.

The clinician decided whether and when to recommend surgery to close the hole and improve VA. All cases involved 23- or 25-gauge microincision pars plana vitrectomy, complete posterior vitreous separation, internal limiting membrane and epiretinal membrane peeling, long-acting gas or silicone oil tamponade, and face-down position.

The posttreatment OCT scan at the specified follow-up interval was analyzed to document the status of MH closure after the first surgery. For this study, the post-surgery anatomical outcomes were divided into two categories as suggested by Kang [12]: (1) Closed MH, where the MH showed type 1 closure without foveal neurosensory retinal defect; and (2) Open MH, where the MH remained unchanged or showed type 2 closure with foveal neurosensory retinal defect even after surgery.

The primary goal of this study was to compare anatomical and functional outcomes of MH after surgery for different aetiologies. Thus, the eyes were divided into two groups: idiopathic or primary MH and non-idiopathic or secondary MH.

The local Institutional Review Board and Ethics Committee approved the study (C-2024-03-003). During registration, patients consented for research participation and publication of their clinical and imaging findings after ensuring to protect their identities.

### Statistical analysis

The statistical analysis was done using GraphPad prism v10.2.3.403 for Windows, developed by GraphPad Software in San Diego, California, USA and available at www.graphpad.com. After determining the data sets’ normality distribution with the Shapiro-Wilk normality test, non-parametric statistical tests were performed. The VA was calculated using Snellen’s scale and converted to ETDRS letters using 85 + 50 x log (Snellen fraction) for analysis. To simplify reading, the change in ETDRS letters after surgery was converted into Snellen lines using a simple formula: five ETDRS letters equal one Snellen line. Categorical variables were expressed as numbers and percentages, while quantitative variables were expressed either as median with interquartile range or mean with standard deviation. The Mann Whitney U test and Wilcoxon matched pairs signed rank test were used to compare quantitative data from two unpaired and two paired groups, respectively. To compare two unpaired groups with categorical data, Fisher’s exact test was used. *P*-values < 0.05 suggested statistical significance.

## Results

This study included 348 eyes from 339 MH patients who met the criteria. Male-to-female ratio was 134:205. The mean age of study participants was 67.32 ± 8.64 years. Secondary MHs were found in 40 (11%) eyes and idiopathic MH in 308 (89%) eyes. Secondary MH was most often caused by blunt ocular trauma in 21 (53%) eyes and pathological myopia in 9 (23%) eyes. At presentation, 231 (66%) phakic eyes had crystalline lenses, 116 (33%) pseudophakic eyes, and one (1%) aphakic eye. The median pre-operative VA for all study eyes was 46 ETDRS letters, ranging from 35 to 55. MH surgery was performed in 181 (52%) eyes without cataract surgery and 167 (48%) eyes with clinically and visually significant cataract at the same time. Table 1 shows the other relevant ocular characteristics and pre-operative MH indices of the study cases. A MH was closed with type 1 closure in 300 (86%) eyes after surgery, and the median VA improvement was 15 ETDRS letters, a 3-line Snellen VA chart improvement.

**Table 1:** Demographic and ocular findings of the entire study cohort:

Variable		Value
Number of eyes (*n*)		348
Number of patients (*n*)		339
Age (Mean, SD)		67.32 ± 8.635
Gender (M: F)		134: 205
Cause of MH	Idiopathic (*n*, %)	308 (89)
	Secondary (*n*, %)	40 (11)
	Ocular trauma (*n*, %)	21 (53)
	Pathological myopia (*n*, %)	9 (23)
	Post-vitrectomy (*n*, %)	3 (8)
	PDR with macular TRD (*n*, %)	3 (8)
	PFT (*n*, %)	1 (3)
	Deroofing of CME (*n*, %)	2 (5)
	RD due to MH (*n*, %)	1 (3)
Lens status	Phakic (*n*, %)	231 (66)
	Pseudophakic (*n*, %)	116 (33)
	Aphakic (*n*, %)	1 (1)
Prefoveal PVD on OCT	Completely detached (*n*, %)	143 (41)
	Partially detached (*n*, %)	68 (20)
	Not visible (*n*, %)	137 (39)
OCT features	ERM (*n*, %)	115 (33)
	Intraretinal cystic spaces (*n*, %)	343 (99)
	RPE reflectivity at the fovea (*n*, %)	320 (92)
OCT based macular hole indices	Maximal basal diameter (microns) [Mean, SD]	1020 ± 327.7
	HFF [Mean, SD]	0.75 ± 0.19
	MHI [Mean, SD]	0.91 ± 0.69
	THI [Mean, SD]	0.20 ± 0.69
	DHI [Mean, SD]	0.09 ± 0.20
	Macular hole area (mm^2^) [Mean, SD]	0.28 ± 0.11
Surgery	Combined cataract + MH surgery (*n*, %)	167 (48%)
	MH surgery alone (*n*, %)	181 (52%)
Pre-operative visual acuity in ETDRS letters (Median, IQR)		46 (35–55)
Post-operative visual acuity in ETDRS letters (Median, IQR)		61 (46–70)
Change in visual acuity in ETDRS letters (Median, IQR)		15 (6–24)
Change in visual acuity in Snellen lines (Median, IQR)		3 (1–5)
Post-surgery MH status	Closed (*n*, %)	300 (86)
	Open (*n*, %)	48 (14)

Abbreviations: SD – standard deviation, M – male, F – female, TRD – tractional retinal detachment, PFT – parafoveal telangiectasia, CME – cystoid macular edema, RD – retinal detachment, MH – macular hole, PVD – posterior vitreous detachment, OCT – optical coherence tomography, ERM – epiretinal membrane, RPE – retinal pigment epithelium, HFF – hole forming factor, MHI – macular hole index, THI – tractional hole index, DHI – diameter hole index, ETDRS – Early Treatment Diabetic Retinopathy Study, IQR – inter-quartile range

Demographics, pre-operative ocular and imaging characteristics, and post-operative anatomic and visual outcomes were compared between idiopathic and secondary MH groups (Table 2). The age and gender distributions of MH cases differed significantly between groups (*p* < 0.05). In this study, idiopathic MH patients were older than secondary MH patients (mean: 68.26 vs. 60.13 years; *p* = 0.001) and more common in women (63% vs. 40%; *p* = 0.005). Only the maximal basal diameter (mean: idiopathic MH = 1005 ± 329.9 vs. secondary MH = 1129 ± 362.2; *p* = 0.031) and diameter hole index (mean: idiopathic MH = 0.09 ± 0.20 vs. secondary MH = 0.04 ± 0.13; *p* = 0.026) of the OCT-based MH indices showed a significant difference between the two groups. Pre-operative VA was comparable between the two groups, but post-operative visual outcomes showed statistically significant differences, with the idiopathic MH group showing better VA improvement (median: 61 ETDRS letters vs. 55 ETDRS letters; *p* = 0.001). The Wilcoxon matched pairs signed rank test compared VA changes before and after MH surgery in both idiopathic (median: pretreatment = 46 ETDRS letters vs. posttreatment = 61 ETDRS letters; *p* = < 0.001) and secondary (median: pretreatment = 43 ETDRS letters vs. posttreatment = 55 ETDRS letters; *p* = < 0.001) MH groups. VA improved significantly in both MH groups after surgery. The idiopathic MH group had significantly better post-surgery anatomical outcomes showing MH closure than the secondary MH group. A greater proportion of secondary MH cases did not close after surgery than idiopathic MH (33% vs. 11%, *p* = 0.001).

**Table 2:** Comparisons between idiopathic and secondary full-thickness macular holes:

Groups (Eyes, Patients)		Idiopathic MH (*n* = 308/300)	Secondary MH (*n* = 40/39)	*P* value
Age (Mean, SD)		68.26 ± 6.535	60.13 ± 16.34	0.001
Gender (M: F)		110:190	24:15	0.005
Lens status	Phakic (*n*, %)	207 (67)	24 (60)	> 0.999
	Pseudophakic (*n*, %)	100 (32)	16 (40)	
	Aphakic (*n*, %)	1 (1)	0 (0)	
Prefoveal PVD on OCT	Completely detached (*n*, %)	131 (43)	12 (30)	0.171
	Partially detached (*n*, %)	63 (21)	5 (13)	0.292
	Absent (*n*, %)	114 (36)	23 (58)	0.016
OCT features	ERM (*n*, %)	96 (31)	19 (48)	0.048
	Intraretinal cystic spaces (*n*, %)	305 (99)	38 (96)	0.103
	RPE reflectivity at the fovea (*n*, %)	284 (92)	36 (90)	0.546
OCT based macular hole indices	Maximal basal diameter (microns) [Mean, SD]	1005 ± 329.9	1129 ± 362.2	0.031
	HFF [Mean, SD]	0.75 ± 0.19	0.75 ± 0.19	0.997
	MHI [Mean, SD]	0.88 ± 0.59	1.15 ± 1.18	0.673
	THI [Mean, SD]	0.22 ± 0.73	0.09 ± 0.35	0.127
	DHI [Mean, SD]	0.09 ± 0.20	0.04 ± 0.13	0.026
	Macular hole area (mm^2^) [Mean, SD]	0.28 ± 0.11	0.30 ± 0.13	0.771
Surgery	Combined cataract + MH surgery (*n*, %)	153 (50)	14 (35)	0.091
	MH surgery alone (*n*, %)	155 (50)	26 (65)	
Pre-operative visual acuity in ETDRS letters (Median, IQR)		46 (36–55)	43 (20–50)	0.236
Post-operative visual acuity in ETDRS letters (Median, IQR)		61 (50–70)	55 (40–65)	0.001
Change in visual acuity in ETDRS letters (Median, IQR)		15 (6–24)	11 (0–20)	0.035
Change in visual acuity in Snellen lines (Median, IQR)		3 (1–5)	2 (0–4)	0.036
Macular hole closure status	Closed (*n*, %)	273 (89)	27 (67)	0.001
	Open (*n*, %)	35 (11)	13 (33)	

Abbreviations: SD – standard deviation, M – male, F – female, MH – macular hole, PVD – posterior vitreous detachment, OCT – optical coherence tomography, ERM – epiretinal membrane, RPE – retinal pigment epithelium, HFF – hole forming factor, MHI – macular hole index, THI – tractional hole index, DHI – diameter hole index, ETDRS – Early Treatment Diabetic Retinopathy Study, IQR – inter-quartile range

We compared VA differences between MH surgery with and without cataract surgery in both idiopathic and secondary MH groups to better understand how cataract surgery improves visual outcomes after MH surgery (Table 3). The table shows that cataract and MH surgery did not improve VA in both study groups (*p* > 0.05).

**Table 3:** Effect of simultaneous cataract and full-thickness macular hole surgery on post-operative visual outcomes:

Visual acuity in	Surgery	Idiopathic MH	Secondary MH	*P*# value
ETDRS letters (Median, IQR)	Combined cataract + MH surgery (*n* = 167)	15 (6–24)	11 (-1–17.75)	0.25
	MH surgery alone (*n* = 181)	15 (6–24)	10.5 (3-20.25)	0.077
	P* value	0.565	0.86	
Snellen lines (Median, IQR)	Combined cataract + MH surgery (*n* = 167)	3 (1–5)	2 (-0.25- 3.5)	0.196
	MH surgery alone (*n* = 181)	3 (1–5)	2 (0.75–4)	0.09
	P* value	0.547	0.708	

Abbreviations: MH – macular hole, ETDRS – Early Treatment Diabetic Retinopathy Study, IQR – inter-quartile range, P* - *p*-value calculated between cases undergoing simultaneous cataract and macular hole surgery and cases undergoing macular hole surgery alone, P# - *p*-value calculated between operated cases of idiopathic and secondary macular holes

## Discussion

There were several key findings from this study. Secondary MH comprised 10% of surgical MH cases. Regardless of the cause, MH surgery led to an average 3-line improvement in VA on the Snellen’s reading chart and a closure rate of ∼ 86%. Combining cataract and MH surgery did not improve visual outcomes in either group. The demographics, pre-operative OCT findings, MH indices, and post-operative functional and anatomical outcomes of primary and secondary MH patients differed significantly. Secondary MH surgery resulted in a lower MH closure rate and less VA improvement than idiopathic MH. Nearly two-thirds of secondary MH surgeries closed the hole, improving VA by two lines on the Snellen’s chart.

An idiopathic MH is caused by progressive foveal vitreous traction, also known as vitreomacular traction, which typically affects the elderly [2, 3]. As a result, idiopathic MH is a disease of the elderly females, most commonly affecting those in their sixth to seventh decades [13, 14]. On the other hand, secondary MHs are most commonly caused by ocular trauma in younger male patients [15]. This explains the statistically significant difference in age and gender distribution observed between our two groups of cases in the study.

In this study, we discovered that the foveal PVD on OCT was not seen in a statistically significant proportion of cases with secondary MH. We also discovered that of the many MH measurements and indices computed in this study, only the maximal basal diameter of the MH and the diameter hole index differed between the two groups of cases. Huang et al. observed similar findings when comparing the presurgical OCT characteristics of full-thickness traumatic and idiopathic MHs [10]. These OCT findings highlight the fact that the pathogenesis of MH formation differs significantly between the two types of cases, with idiopathic MHs developing mainly due to antero-posterior vitreous traction and secondary MHs occurring mainly due to lateral tangential forces exerted on the fovea.

With the most recent advances in surgical instrumentation and techniques for the management of MHs, vitrectomy surgery is the first and most preferred method of managing idiopathic MHs of any size [8, 16]. On the other hand, the standard management strategy for secondary MHs is usually observation and deferring the vitrectomy surgery. Surgery is usually reserved for secondary MHs that do not close spontaneously, even after the primary cause has been addressed and when there is a chance that VA will improve after the MH has successfully closed following surgery [7]. Thus, in our study, we found that only 10% of the operated MH cases belonged to the secondary MH group. Zhou et al. published a systematic review and meta-analysis that suggested a positive anatomical and functional outcome in cases of traumatic MHs undergoing surgery [9, 17]. The current study found similar results, with nearly two-thirds of secondary MH cases achieving type 1 closure and a two-line visual gain after surgery. However, the VA gains in the secondary MH group did not match those seen in the idiopathic MH group. Despite this, surgery for secondary MH cases needs to be considered early in the treatment plan.

The surgical steps for treating any type of MH are nearly identical. Irrespective of the cause of the MH, the primary objectives involved in MH surgery include releasing the different tractional forces acting on the fovea by inducing the posterior vitreous separation completely and peeling the internal limiting membrane (ILM) and overlying epiretinal membrane to the extent possible, blocking the MH by injecting intraocular gas or silicone oil tamponade and maintaining prone position, and allowing the reparative glial tissue to proliferative over a scaffold provided mainly by the tamponading agent and sometimes by the use of additional adjuvants [18]. As a result, anatomical MH closure rates should be comparable in the idiopathic and secondary MH groups. However, in this study, we discovered that the surgical anatomical outcomes for type 1 MH closure rates were lower in the secondary MH group than in the idiopathic MH group. Anatomical outcomes following MH surgery are determined by the morphology of the MH. In our study, we found a significantly larger basal diameter of the MH in the secondary MH group. The larger basal diameter of the MH could be attributed to tangential tractional forces centrifugally pulling the retina at the macula, as well as the longer duration of the untreated MH due to deferred vitreous surgery. Studies have found similar results that MHs caused by high myopia and ocular trauma have poorer anatomical outcomes than idiopathic MHs [15, 19]. Despite having a lower MH closure rate in secondary MH cases than in idiopathic MHs, surgery is still recommended to increase the chances of early MH closure and improve VA faster.

The improvement in VA following MH surgery is determined by pre-operative factors such as the presenting VA, cause, morphology, and stage of the MH, as well as intraoperative dye use and, finally, the post-operative health of the RPE [20]. According to studies, there is a positive correlation between pre-operative and post-operative VA after MH surgery. Additionally, a negative correlation has been found between the post-operative VA and the pre-operative stage of the MH and the area of RPE damage caused by the use of intraoperative dyes such as indocyanine green and brilliant blue G (BBG) [21–24]. The VA outcomes differ depending on the MH type. Compared to MHs without high myopia, high myopic MH has limited postoperative visual outcomes. Wu and Kung found that in a group with high myopia, the mean logMAR VA improved from 0.92 to 0.63, whereas in a group without high myopia, the improvement was from 1.02 to 0.48 [19]. In the current study, we found a significant difference in the amount of VA gain between idiopathic and secondary MH cases. Cases with idiopathic MHs showed greater vision improvement than cases with secondary MHs, which supports the findings of Wu and Kung [19]. Depending on the stage of the MH, the average VA has been reported to improve to 20/50 for stage 2 holes, 20/110 for stage 3 holes, and 20/145 for stage 4 [21]. In the literature, poor VA outcomes have been reported as a result of RPE damage caused by the use of toxic dyes such as indocyanine green and BBG combined with endolight usage [21, 23, 24]. BBG has been shown to provide better visual outcomes after ILM peeling than indocyanine green. According to Williamson and Lee, postoperative VA was 20/100 for patients who underwent surgery with indocyanine green, and 20/70 for patients who underwent surgery with BBG [21]. The secondary MH group showed a statistically significant 2-line improvement in VA, but not as much as the idiopathic MH group, which showed a 3-line improvement in the current study.

This study represents one of the largest single-center analyses comparing idiopathic and secondary MHs in terms of demographic distribution, pre-operative OCT findings, and post-operative outcomes. It underscores the value of surgical intervention in improving closure rates and VA for both types of MH, with findings that are directly applicable to clinical practice without reliance on complex statistical models. However, several limitations should be noted. The retrospective design precluded the assessment of the natural history of idiopathic and secondary MHs, as only surgical cases were included. Additionally, the relatively small sample size of secondary MH cases limited our ability to draw statistically significant conclusions about different subtypes. Further research with larger cohorts is necessary to better characterize secondary MH subgroups. The study also did not evaluate potential surgical variables, such as surgeon experience, ILM peeling techniques, or endotamponade type, which could influence outcomes. Despite these limitations, our findings provide practical insights for clinicians, helping them to counsel patients on the expected anatomical and visual prognosis after MH surgery, regardless of etiology.

To summarise, non-idiopathic or secondary MHs differ from idiopathic or primary MHs in terms of demographic distribution, pathogenesis, clinical findings, and surgical outcomes. To achieve good anatomical and functional results, surgery for a MH should be the preferred treatment option, regardless of its type.

## Data Availability

No datasets were generated or analysed during the current study.

## Declarations

### Presentation at meeting

No.

### Animal research

“This article does not contain any studies with animals performed by any of the authors.”

## Abbreviations

MH: Macular hole
RPE: Retinal pigment epithelium
OCT: Optical coherence tomography
VA: Visual acuity
BBG: Brilliant blue G
ILM: Internal limiting membrane
